# Supplementary material for: A web-database of mammalian morphology and a reanalysis of placental phylogeny
Source: BMC Evol Biol. 2007 Jul 3;7:108. doi: 10.1186/1471-2148-7-108 (PMC1941728; doi:10.1186/1471-2148-7-108)
Supplement: Additional File 1 — Website data: The 196 characters first described in [17], along with the supplementary data indicated below, have been joined into a number of HTML files, all of which are linked to the "morphsite_bmc07.html" index page. The embedded links to the supporting files are functional, independent of PC or platform, provided that the directory structure remains intact (i.e., all files remain in a single folder, no subdirectories). Nexus files: Datafiles containing the morphological, indel, and aligned DNA sequence data, linked from the "morphsite_bmc07.html" index page. Table 1. Changes to the multiple sequence alignment of [9]. Table 2. Qualitative character-state corrections to the morphology matrix of [17]. Table 3. Summary of discrepancies between Appendices 1 and 2 of [17]. [file 1471-2148-7-108-S1.zip › asher-BMC07-website/cranium.html]

asher-characterBank


|  |  |  |
| --- | --- | --- |
| Cranium |  | This work is licensed under a   Creative Commons License. |
| Middle Ear  1 stapes obturator foramen  ;  0 large 1 small-abs,    2 petrosal-squamosal joint  ;  0 flat 1 ball-socket 2 minimal contact 3 tubular,   3 squamosal external auditory meatus (EAM) ;  0 no approximation ventral EAM 1 squamosal ventral EAM,   4 fenestra cochleae ;  0 present 1 absent,   5 fenestra cochleae orientation  ;  0 posterolat 1 posterior,   6 jugular foramen  ;  0 similar fenestr coch 1 larger fenestr coch,   7 crista interfenestralis  ;  0 abs 1 pres,   8 width basal cochlea  ;  0 <20% basicran width 1 >20% basicran width,   9 anterior promontory  ;  0 anteriorly smooth 1 projects,   10 petrosal canals ;  0 absent 1 present,   11 transpromontorial sulcus ;  0 pres 1 abs,   12 stapedial sulcus  ;  0 pres 1 abs,   13 piriform fenestra ;  0 absent 1 present,   14 petrosal pachyostosis  ;  0 light 1 dense,   15 tegmen tympani pneumatization ;  0 laminar 1 air cells,   16 mastoid pneumatization ;  0 laminar 1 air cells,   17 tegmen tympani process ;  0 flat 1 projects,   18 epitympanic recess ;  0 continuous middle ear 1 separate,   19 basisphenoid bulla ;  0 reduced 1 present,   20 alisphenoid bulla ;  0 reduced 1 present,   21 basioccipital bulla  ;  0 absent 1 shields bulla medially,   22 caudal tympanic process petrosal;  0 reduced 1 shields posterior bulla,  23 rostral tympanic process petrosal;  0 reduced 1 present,   24 ectotympanic;  0 phaneric 1 occluded,   25 ectotympanic shape;  0 ringlike 1 expanded,   26 ectotymanic involucrum;  0 abs 1 pres,   27 external auditory meatus shape  ;  0 ovoid 1 ventral extension,   28 tympanic annulus ;  0 supports membrane 1 conical ligament,   29 entotympanic  ;  0 small-abs 1 present, | Basicranium  30 mastoid exposure  ;  0 exposed 1 occluded,   31 petromastoid exposure   ;  0 posterior 1 lateral,   32 foramen ovale ;  0 within alisph 1 post alisph 2 squamosal,   33 alisphenoid canal   ;  0 present 1 absent,   34 alisphenoid canal position ;  0 lateral sphenorbital fissure 1 confluent sphenorbital fissure,   35 maxilla-squamosal connection  ;  0 unconnected 1 connected ventral sphenorbital fissure  36 ectopterygoid  ;  0 single 1 dual,   37 basisphenoid pit ;  0 absent 1 present,   38 posterior lacerate foramen  ;  0 narrow 1 anteroposteriorly elongate,   39 hypoglossal foramen ;  0 abs 1 single 2 multiple,   40 ventral basisphenoid  ;  0 exposed 1 covered by vomer 2 covered by pterygoids, | Braincase 41 petromastoid exposure in braincase  ;  0 exposed 1 excluded,   42 subarcuate fossa ;  0 present 1 flat 2 semicircular canals reduced,   43 dorsum subarcute fossa  ;  0 closed 1 open,  44 tentorium cerebelli   ;  0 absent 1 divides braincase,   45 sinus canal  ;  0 absent 1 present,   46 dorsum sellae ;  0 weak 1 prominent,   47 crista galli   ;  0 flat 1 dorsally projects 2 cribriform plate abs, |
| Orbitotemoporal Region  48 foramen rotundum ;  0 confluent sphenorbital fissure 1 distinct sphenorbital fissure,   49 alisphenoid spine  ;  0 flat 1 anteriorly projects,   50 optic foramen;  0 absent 1 distinct,   51 optic foramen size   ;  0 caliber as V 1 caliber smaller V,   52 suboptic foramen  ;  0 absent-hidden 1 present,   53 interorbital fenestra  ;  0 absent 1 connects orbits,   54 ethmoid foramen ;  0 present 1 absent,   55 tuber maxillaris  ;  0 absent 1 dorsal ridge in orbit,   56 Glenoid pos  ;  0 even petrosal 1 dorsal petrosal,  57 glenoid shape   ;  0 transverse 1 anteroposteriorly elongate 2 dual,   58 postglenoid foramen  ;  0 separate glenoid 1 at glenoid,   59 postglenoid process  ;  0 absent 1 present 2 pres-AP,   60 ento-postglenoid processes  ;  0 separate 1 connected,   61 entoglenoid process  ;  0 no-lat jaw support 1 post support,   62 zygomatic arch ;  0 complete 1 incomplete 2 squamosal-frontal,   63 jugal in glenoid ;  0 reaches glenoid 1 reduced anterior to glenoid,   64 anterior jugal ;  0 anterior edge orbit 1 lateral orbit,   65 zygomatic process squamosal ;  0 robust dorsolaterally 1 narrow laterally,   66 postorbital bar ;  0 reduced 1 surrounds 90% orbit,   67 postorb septum ;  0 absent 1 jugal-alisphenoid contact,   68 interorbital distance  ;  0 separate 1 convergent,   69 facial lacrimal process  ;  0 <= orbital process 1 large facial process,   70 orbital lacrimal process ;  0 flat 1 projects into orbit,   71 lacrimal foramen  ;  0 present, 1 not exposed anterior orbit,  72 lacrimal foramen opening ;  0 posterior 1 lateral,   73 frontal in orbit ;  0 reduced contact palatine 1 broad contact palatine,   74 jugal in medial orbit  ;  0 jugal restricted 1 jugal-parietal contact,   75 maxilla in medial orbit  ;  0 reduced 1 extensive, | Rostrum  76 premaxilla-frontal contact ;  0 absent 1 present 2 present over maxilla,  77 anterior nasals  ;  0 fused with premaxilla 1 projecting,   78 posterior nasals  ;  0 tapered 1 broad 2 horns,   79 metopic suture  ;  0 unfused 1 fused,   80 maxilla medial to zygoma  ;  0 solid 1 fenestrated 2 trabeculated,  81 infraorbital canal length  ;  0 long 1 short,   82 infraorbital canal anterior foramen  ;  0 small 1 similar ext nares,   83 orbit position to alveolar maxilla ;  0 anterior 1 dorsal 2 posterior,  84 external nares ;  0 anterior rostrum 1 dorsal cheek teeth 2 posterior rostrum, | Palate  85 postpalate spine  ;  0 flat 1 prominent,   86 posterior hard palate to toothrow  ;  0 posterior 1 even 2 anterior,   87 palatal notch  ;  0 closed medial M3 1 open medial M3,   88 posteroventral maxilla ;  0 at toothrow 1 posterior toothrow ventrum pterygoid,   89 palatal premaxilla exposure ;  0 abs-alveoli only 1 small 2 half palate,   90 incisive foramen presence;  0 present 1 absent,   91 incisive foramen number ;  0 dual opening palate 1 single opening palate,   92 incisive foramen shape  ;  0 oval 1 elongate,   93 palatine fenestrae  ;  0 solid 1 posterior 2 anterior |
